# The Use of Telerehabilitation Technologies for Cardiac Patients to Improve Rehabilitation Activities and Unify Organizations: Qualitative Study

**DOI:** 10.2196/10758

**Published:** 2018-11-19

**Authors:** Birthe Dinesen, Helle Spindler

**Affiliations:** 1 Laboratory for Welfare Technology – Telehealth & Telerehabilitation Sensory-Motor Interaction, Department of Health Science and Technology Faculty of Medicine, Aalborg University Aalborg East Denmark; 2 Department of Psychology and Behavioural Sciences Aarhus University Aarhus Denmark

**Keywords:** telerehabilitation, heart diseases, workflow, cooperation, professional practice, community of practice

## Abstract

**Background:**

Cardiovascular disease is a leading cause of death globally causing 31% of all deaths worldwide. The Danish health care system is characterized by fragmented delivery of services and rehabilitation activities. The Teledialog Telerehabilitation Program for cardiac patients was developed and tested to rectify fragmentation and improve the quality of care. The Teledialog program was based on the assumption that a common communication platform shared by health care professionals, patients, and relatives could reduce or eliminate the fragmentation in the rehabilitation process and improve cooperation between the health professionals.

**Objective:**

This study aimed to assess the interorganizational cooperation between health care professionals across sectors (hospitals, municipal health care centers) in a cardiac telerehabilitation program.

**Methods:**

Theories of networks between organizations, the sociology of professions, and the “community of practice” approach were used in a case study of a cardiac telerehabilitation program. A triangulation of data collection techniques were used including documents, participant observation (n=76 hours), and qualitative interviews with healthcare professionals (n=37). Data were analyzed using NVivo 11.0.

**Results:**

The case study of cooperation in an interorganizational context of cardiac telerehabilitation program is characterized by the following key themes and patterns: (1) integrated workflows via a shared digital rehabilitation plan that help integrate workflow between health care professions and organizations, (2) joint clinical practice showed as a community of practice in telerehabilitation developed across professions and organizations, and (3) unifying the organizations as cooperation has advanced via a joint telerehabilitation program across municipalities and hospitals.

**Conclusions:**

The Teledialog Telerehabilitation Program was a new innovative cardiac program tested on a large scale across hospitals, health care centers, and municipalities. Assessments showed that the Teledialog program and its associated technologies helped improve interorganizational cooperation and reduce fragmentation. The program helped integrate the organizations and led to the creation of a community of practice. Further research is needed to explore long-term effects of implementation of telerehabilitation technologies and programs.

**Trial Registration:**

ClinicalTrials.gov NCT01752192; http://clinicaltrials.gov/ct2/show/NCT01752192 (Archived by WebCite at http://www.webcitation.org/6yR3tdEpb)

## Introduction

Cardiovascular disease (CVD) is a leading cause of death on the global scale [1]. It is estimated that 17.5 million people died from CVD in 2012, equivalent to 31% of all global deaths [1]. Fortunately, CVD can be reduced by addressing behavioral risk factors such as unhealthy diet, obesity, physical inactivity, tobacco use, and excessive alcohol consumption. Cardiac rehabilitation (CR) programs include interventions such as exercise and patient education on risk factors, encouraging the patient to pursue and maintain a healthy lifestyle. Nevertheless, effective implementation of CR following CVD has been inadequate, with participation rates below 50% over recent decades, despite international recommendations [2-5]. A review of the literature highlights several factors that impede patients’ participation in CR programs including (1) inadequate access to health care services, (2) fragmentation of the organization of rehabilitation efforts between hospitals and local health centers, (3) the patient's lack of motivation and inability to manage their disease, (4) lack of individualized rehabilitation programs, and (5) transport difficulties to the clinic [4,6,7].

The rehabilitation of cardiac patients has evolved from a formerly hospital-based system to a cooperative arrangement that brings together hospitals, health centers, and municipalities [8,9]. The CR of patients is now divided into more specialized rehabilitation activities carried out at hospitals and general rehabilitation activities carried out under the guidance of health care centers linked to municipalities [8,9]. From a comparative international perspective, health care systems are characterized by fragmentation of health care services and rehabilitation activities [10-15]. This fragmentation generates and reproduces knowledge gaps between health care professionals in hospitals and municipalities, loss of information regarding the patient’s status after they are discharged from hospital and referred to a health care center, and a lack of cross-sector coordination in specific rehabilitation activities [14]. This fragmentation process has been ongoing for years, and in a recent survey by the Danish Heart Association, cardiac patients stated that fragmentation remains an organizational barrier for their successful CR [16]. A promising new solution to meet the challenges of this fragmentation is the use of telerehabilitation (TH) for cardiac patients. The term TH is defined as the delivery of rehabilitation services via information and communication technologies [17]. A review of alternative models of CR points out that there is no need to rely only on hospital-based strategies. Community and home-based programs can be used to design a more individualized rehabilitation that can be tailored to the patient’s specific needs and abilities [18-20].

Evaluations of cardiac telerehabilitation (CTH) programs conclude that studies tend to be heterogeneous regarding patients, intervention, use of technologies and outcome measures. Moreover, CTH programs often lack nutritional counseling or psychosocial management [21,22]. Studies focusing solely on exercised-based CTH have been shown to be at least as effective as center-based rehabilitation for improving functional capacity and reducing CVD risk factors [23]. At present, we have found no studies that have focused on the impact of TH technologies on coherence within the cardiac rehabilitation process or cooperation across sectors (ie, between health care professionals in hospitals and health centers in municipalities).

We define “cooperation” as an arrangement in which two or more parties who might otherwise compete with each other engage in a voluntary and mutually beneficial exchange [24]. Cooperation across sectors is desirable because (1) it helps avoid fragmentation, (2) it ensures continuity in information and communication flow in the patient care processes, (3) it brings together complementary competencies between health care professionals, (4) it reduces the potential for adverse events, and (5) it generally secures the quality of care [14,15,25].

This study aims to explore interorganizational cooperation between health care professionals across hospitals and municipalities as it occurs within a single program, the Teledialog Telerehabilitation Program (TTP), and its associated technologies.

## Methods

### Design

This study is a substudy carried out within the main TTP. The descriptive case study, provided by Robert Yin [26] is the overall method chosen for this study. A case study is defined as “an empirical inquiry that investigates a contemporary phenomenon (the ’case’) in depth and within its real-world context, especially when the boundaries between phenomenon and context may not be clearly evident” [26].

### Description of Sampling

In phase I, from November to December 2012, health care professionals (nurses, a physician, and physiotherapist) were selected based on 2 criteria: (1) they were working within the team of CR staff at a hospital or health care center for more than a year and (2) they were involved in practical CR. In phase II, from December 2013 to January 2014, health care professionals were selected for interviews based on having been directly involved with cardiac patients participating in the TTP at a hospital, health care center or call center.

### Presentation of Context in a Case Study

**Figure 1:**
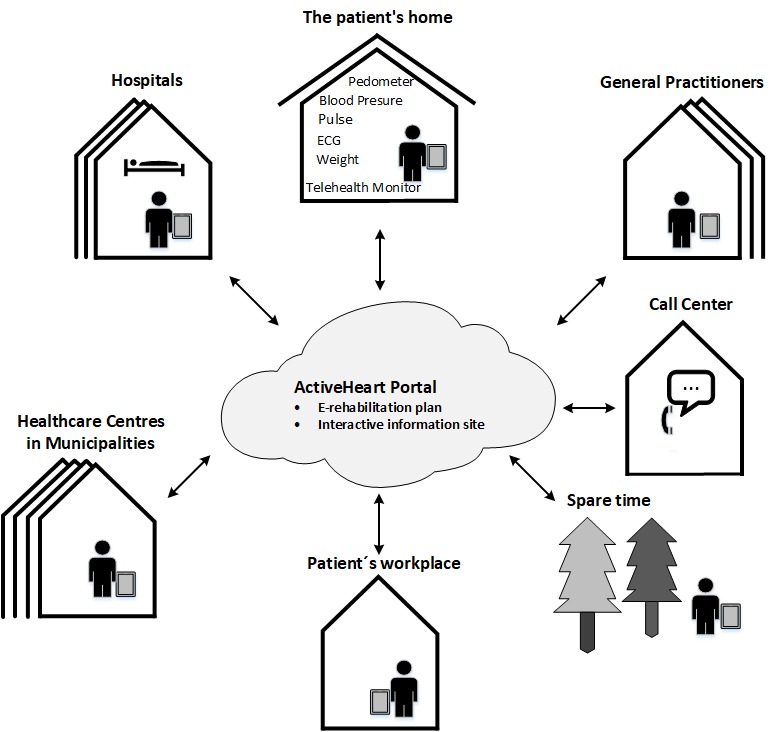
The Teledialog Telerehabilitation Network. The small grey square in each “house” represents a mobile device (eg, tablet) that patients have close by and use to transmit information or communicate with health care personnel from home, work or during leisure activities. ECG: electrocardiography.

The TTP was developed from May 2011 to March 2012. The program was based on user-driven innovation [27] in workshops involving a range of participants including (1) health care professionals from hospitals and health care centers, (2) cardiac patients, (3) relatives, (4) representatives from companies, and (5) researchers from disciplines such as nursing, medical engineering, psychology and organizational sociology. Participants in the CTH program were a cardiology ward at a regional hospital, a thoracic ward at a university hospital, 4 health care centers located in 2 municipalities and a call center. The Teledialog Network is centered around a Web portal called “ActiveHeart” (see Figure 1).

The target group in this study consisted of patients diagnosed with heart failure, myocardial infarction, angina pectoris, and who had undergone coronary artery bypass surgery. The overall aim of the TTP was to develop a more individualized rehabilitation process, avoid organizational fragmentation and facilitate coherence in the rehabilitation process. Within the TTP, the rehabilitation program was carried out in close collaboration between the cardiac patients, hospitals, health care centers and a call center between 2012 to 2014. The cardiac patients tested the TH program for 12 weeks (see Table 1 for project overview). A video of the Teledialog project is provided in Multimedia Appendix 1.

Each patient was interviewed individually before discharge in order to determine their specific rehabilitation needs and type of rehabilitation program (hospital, health care center, or call center). An individualized rehabilitation plan was then designed with the patient, following current guidelines for cardiac recommendations as developed by European Association of Cardiovascular Prevention and Rehabilitation [28] and the Danish Health Agency [8,9].

**Table 1:** Overview of the technologies used in the Teledialog project.

Technology/device	Function
Tablet	Patients used an Android tablet to access data
ActiveHeart.dk	An interactive portal that functions as a toolbox for cardiac patients Site contains information on a range of relevant rehabilitation issues (medicine, smoking, mental well-being, diet, and physical exercises) Patients could access the information on a 24/7 basis Information was communicated in text, video, and audio and designed to suit patients’ preferred style of information-seeking
Shared Care Platform (e-rehabilitation plan)	Platform for everyday use between health care professionals, patients, and relatives Provides an overview of patient data including medications, goal, and plan for rehabilitation, diary, hospital or health care center appointments and monitored values (blood pressure, pulse, weight, and steps)
CareConnect	A data platform for integrating and connecting the different project systems CareConnect received data from Danish national standards, MyMedic, Fitbit, and the e-rehabilitation plan
Triage Manager	Health care professionals used this module at hospitals, health care centers, and a call center Administered data on the patients being monitored
Telehealth monitor	Data was transmitted using MyMedic to transfer data via a mobile internet connection to a central server Used to transmit data from devices (eg, the sphygmomanometer, digital weight scale, and electrocardiography)
Sphygmomanometer	Meter was paired with the telehealth monitor in advance
Digital weight scale	Weight scale was paired with the telehealth monitor in advance
Fitbit Ultra	Digital pedometer that enabled patients to view steps taken in their e-rehabilitation plan

All patients received training in the use of the various rehabilitation devices, navigation of the ActiveHeart Web portal, and in interpreting and using the e-rehabilitation plan (see Table 1). A physician prescribed the frequency with which the patient needed to measure their blood pressure, pulse, and weight (usually twice a week). Steps were measured every day. All data were transmitted to the e-rehabilitation plan database via a secure transmission line. Nurses at a call center recalibrated the measured values so that the health care professionals in the municipalities could then assess the monitored values each week. During the implementation of the TTP, the health care professionals held 5 meetings of 2 hours each in which they discussed TH issues for the cardiac patients and how to best cooperate and coordinate their activities.

### Theory

Theories of networks between organizations [24], the sociology of professions [29], and learning theory [30] constitute the tripartite theoretical framework for this case study.

A network is defined as “the basic social form that permits interorganizational interactions of exchange, concerted action, and joint production. Networks are unbounded or bounded clusters of organizations that, by definition, are non-hierarchical collectives of legally separate units” [24]. Described by Abbott [29], the sociology of professions has been applied in order to help focus on the dynamics and interplay between health care professionals from hospitals, call center, and health care centers. The perspective focuses on professional work, social relations, and internal struggles between occupational groups in an interprofessional context. Finally, learning theory, described in the work of Wenger [30], has been applied in order to highlight the emergence of “communities of practice.” Wenger defined “communities of practice” as groups of people who share a concern or passion for something they do and who interact on a regular basis*.* The strategy here was to investigate how the technologies used in the Teledialog network affected the learning process among the participants. Central themes from the theoretical framework have been applied in the observation and interview guides used in the data collection process (see Multimedia Appendix 1 and Multimedia Appendix 2).

### Data Collection Techniques in the Case Study

In this study, a triangulation of data collection techniques was used in order to validate the data.

### Document Analysis

As background for the descriptive case study, documents and reports on the organization of rehabilitation activities, strategies, and policies within rehabilitation and homepages from hospitals and health care centers were studied. The aim of this background documentation was to (1) obtain intensive knowledge of the context for the case study, such as how conventional rehabilitation had been carried out, (2) division of tasks between health care professionals across sectors, and (3) how communication and information flow between the health care professionals in the interorganizational context.

**Table 2:** Overview of interviewees.

Respondents				Phase I (Nov-Dec 2012), n		Phase II (Dec 2013-Jan 2014), n
**Hospital staff**						
		Nurses		3		4
		Physicians		2		2
		Physiotherapist		1		1
**Health care center staff**						
	Nurses		7		7	
	Physiotherapists		4		4	
Call center staff (nurses)					2	
Total				17		20

### Participant Observation

Participant observation [31] was carried out to observe (1) cooperation between health care professionals in hospitals and municipality health centers and (2) their relations with the patients participating in the TH program. Observations were carried out during meetings, at patient discharge, and during the daily routine work of health professionals across sectors and in interactions with patients and relatives based on an observational guide (Multimedia Appendix 2).

As part of the participant observation, we studied communication between health professionals and patients enrolled in the e-rehabilitation plan. The 3 main themes were (1) planning and coordination of the rehabilitation program, (2) communication among groups of health professionals, and (3) communication between health care staff and patients and relatives.

The 2 authors performed participant observation for a total of 76 hours. Through observations in various settings, data was collected on communication patterns at meetings, problem-solving and interaction between health care professionals carrying out clinical tasks across sectors, and interaction between health professionals and patients. Field notes were taken immediately after the observation had taken place, entered as Microsoft Word files, and then analyzed using the NVivo 11.0 qualitative data analysis program.

### Qualitative Interviews

The two authors of this study conducted semistructured qualitative interviews described by Kvale and Brinkmann [32] with representatives from all health care organizations involved in the TH of the cardiac patients (Multimedia Appendix 3).

The interviews were conducted in 2 phases. During phase I, the health care professionals were asked to describe how they experienced the cross-sectoral co-operation within cardiac rehabilitation. The aim was to obtain a basic understanding of the context of the case study. In phase II, interviewees were asked to explore the interorganizational cooperation within the TTP and specifically, how they experienced cooperation across sectors using the digital platform. The interviews in both phases lasted from 55-90 minutes (see Table 2).

### Data Analysis

A research assistant transcribed all interviews. The transcribed interviews, documents, notes from participant observation were coded using NVivo 11.0 software and analyzed in steps described by Kvale and Brinkman [32]. The data were analyzed using a combination of deductive and inductive strategies. A code tree was designed based on key definitions and concept from the theoretical framework and the interviews. As a first step in formulating the concepts from the respondents, the qualitative interviews were studied and coded by initial impression. This was followed by a rough coding and refined coding based on the reviews of coded data and adjustments. This second step sought to identify key themes and patterns relevant to identifying the participants’ views about cooperation and TH. The final step in the data analysis was an in-depth interpretation that was put in contrast with the participants’ own common sense understandings and motivations. The coding and analysis were carried out by the authors, both of whom have backgrounds in nursing, organizational development, and psychology. To ensure intercoder reliability, the same 2 researchers initially had dialogue and compared codes in order to agree on definitions for subsequent coding, since using a software program to analyze data may decontextualize the analysis of data.

### Ethical Considerations

The Teledialog project was approved by the Danish Ethical Committee (N-20120051), and the project was registered at ClinicalTrials.gov (ClinicalTrials.gov identifier NCT01752192). The study was performed according to the Declaration of Helsinki.

## Results

### Key Themes and Findings

In this section the key themes and findings on interorganizational cooperation in the TTP are presented (see Table 3).

**Table 3:** Key themes and findings from interorganizational cooperation in the Teledialog Telerehabilitation Program.

Key themes	Findings
Integrated workflows	Shared e-rehabilitation plan integrates the workflows between the organizations A new way of communicating and sharing clinical data (blood pressure, pulse, weight, steps, and rehabilitation plan for the patients) between the participating health care organizations The technological platform facilitates interdisciplinary decision-making on rehabilitation issues
Joint clinical practice	A telerehabilitation community of practice emerged Relations between health care professionals was strengthened Knowledge-sharing on rehabilitation issues enhanced Joint vision on telerehabilitation for cardiac patients across sectors and municipalities Patients became collaborators with health care professionals rather than passive clients
Unifying organizations	Joint telerehabilitation program enhanced cooperation across hospitals and municipalities Increased mutual use of health care professionals’ know-how and manpower across municipalities (regarding administrative rules and budgets) and hospitals Staff had increased sense of being a single, unified organization

In the following sections, the key themes and findings are elaborated. Illustrative quotations from interviews with health care professionals are presented in the following. The criteria for selecting the quotations was that they should represent the overall theme or subthemes of the data.

### Integrated Workflows

Health care professionals expressed the view that the e-rehabilitation plan across hospitals and municipalities enabled them to share data on each patient’s rehabilitation program and to communicate with each other and with patients and relatives on a continuing basis. The plan made it possible to establish a high level of coherence and continuity during the entire patient rehabilitation process.

The digital platform makes it possible for us to share data on the patients between hospital and municipalities. Sharing data prevents adverse events and increases the quality of planning for rehabilitation after patients are discharged from hospital. I think we have reduced fragmentation.

Nurse #20, female

By having online access to the same data for a single cardiac patient, the health professionals concluded that the use of the technological platform facilitated interdisciplinary decision-making for the benefit of the patients. In the beginning, the professionals felt that the task was challenging. However, our observational notes showed that after 4 months, the technological platform became an integrated part of the workflow across sectors.

Having access to the same data about a patient makes it possible for us across sectors and professional organizations to carry out interdisciplinary decision-making within rehabilitation…in the beginning it was difficult, but after some time we realized the benefit of doing it [this way].

Nurse #25, female

### Joint Clinical Practice

Health care professionals expressed that their relations were strengthened during their work within the TTP. They met with each other regularly in order to discuss issues within TH.

The meetings we’ve had during the project and the [use of the] digital platform have strengthened relations between our teams.

Physiotherapist #33, female

In the interviews, the health professionals expressed the view that the meetings were effective channels for knowledge-sharing and creating a joint vision for TH of cardiac patients. Observations identified engagement and knowledge-sharing between the health professionals from the cardiology ward and those working in the health centers.

We’ve had the possibilities to exchange knowledge about the challenges of rehabilitation of cardiac patients and to make a joint vision together…I feel like we are working in the same organization.

Nurse #18, female

Not all patients can participate in the telerehabilitation program, so we need to discuss with each other which patients are capable of taking part in the telerehabilitation program.

Nurse #28, female

The professionals stated that most of the patients monitored their data very carefully and engaged actively in their rehabilitation process in order to return to everyday life more quickly. Professionals in the municipalities described the patients as collaborators rather than passive clients.

We feel the patients are becoming more engaged in their own health and rehabilitation because they can see their own data and are part of the telerehabilitation program.

Nurse #30, female

### Unifying Organizations

In geographic terms, the Danish TH program covered 2 hospitals and 4 health care centers in 2 municipalities and a call center. The TH program and its associated technologies made it possible to offer a new joint rehabilitation service on a large scale. The benefits were from pooling resources and know-how, and to offer patients in remote areas the possibility to carry out their rehabilitation in their local community health care centers and in their own homes, thus reducing disruption so as not to disrupt their everyday routines.

By having the digital platform, we can substitute for each other during vacation periods and give patients the same level of service.

Nurse #27, female

Telerehabilitation is a new way of working as a team and of bringing synergy between our disciplines, know-how and manpower and municipalities.

Physiotherapist #35, female

Observational notes showed that at the beginning of the implementation of the program, there was some frustration among the health professionals in figuring out how to cooperate across sectors and implement the new workflows. By the final evaluation, however, the data from interviews and observations showed that these frustrations were no longer present.

The health care professionals expressed that the interorganizational cooperation and use of the joint e-plan enabled them to bridge across professions and organizations, giving them a feeling of being a single, unified organization.

It’s easier for us to communicate via the digital platform. It makes us feel like a single organization, but it’s important to have the meetings face-to-face.

Nurse #31, female

## Discussion

### Principal Findings

The case study of cooperation in an interorganizational context of a cardiac TH program showed (1) the shared digital rehabilitation plan helped increase the level of workflow integration between health care professions and the participating organizations, (2) the joint clinical practice developed into a TH community of practice across professions and organizations, and (3) municipal and hospital organizations became more unified due to their cooperation in the joint TH program.

### Interpreting Findings in the Context of the Wider Literature

The TTP was the joint vision for the systematic network of the participating organizations. Planning and coordinating the individualized rehabilitation processes for the cardiac patients was the prime focus for the health care professionals in the Teledialog Network. Regular meetings between health professionals and the elaboration of individualized e-rehabilitation plans for patients constituted the platform for cooperation, knowledge-sharing, coordination, and joint problem-solving between members of the participating organizations.

The e-rehabilitation plan can be compared to a personal health record (PHR), which is an electronic app where individual patients can access, manage, and share health information with anyone whom they allow [33]. The adoption, acceptance, and use of PHR requires a culture of adaptation, user-friendly technology, and a governance structure [34,35]. The governance structure in the TTP, including content, vision, and distribution of tasks and responsibility among health care professionals across sectors, was negotiated and developed in workshops with health care professionals before implementation of the program. A review of the dynamics of interorganizational collaboration [25] states that if participants are involved issues for discussion are agreed on in the process of planning so that it does not become a barrier. Also, it may explain the positive result of this study. The work by Barlow et al [36] supports these findings by emphasizing that implementing complex innovations in an interorganizational context with many stakeholders requires that all parties have had sufficient opportunity to share views and to have an open dialogue on values. We have not identified any cardiac TH studies that have described such a shared care platform in an interorganizational context.

A community of practice was established across professions and organizations in the Teledialog project. These findings are in alignment with Wenger’s community of practice theory [30]. The development of a community of practice based on a digital platform across sectors has previously been identified among patients with chronic pulmonary diseases and health professionals. The result was that the parties could exchange experiences, stories, and strategies for how to manage rehabilitation in the patient’s homes [37]. The theoretical framework has helped to identify the dynamics of cooperation and learning processes between the health care professionals working across hospitals, sectors, and municipalities within the TTP.

A review by Rolls et al [38] concluded that health care professionals who used social media to develop virtual communities to share domain knowledge often exhibit tribal behaviors between each other with the result being a limitation on knowledge sharing. We did not identify this kind of issue in our study, even though we have utilized the sociology of professions approach as part of our theoretical framework. The same authors highlight the need for further research in order to evaluate the effects of social media on knowledge distribution in clinical practice and, equally important, to assess whether patient outcomes are significantly improved. Busetto et al [39] and Otte-Trojel [40] report findings that support our results concluding that IT can serve as a facilitator for complex interventions within integrated care.

The interorganizational cooperation in the Teledialog project was advanced. Mandell et al [41] highlight the fact that for interorganizational innovations to be successfully implemented in a complex context, management/project management must be made aware of the impact of contextual factors. The important factors are the history of relationships, the relative power of the actors in the network, imposition of rules, impact of political/cultural context and culture of the actors. By using the case study with a triangulation of data collection techniques, our interdisciplinary team was able to bring the contextual factors into the analysis as part of the preparation for the trial. By identifying the factors before implementation of the cardiac TH program, implementation became possible within the time frame and the budget of the project. Moreover, we were able to overcome the factors that typically impede or derail the implementation of eHealth systems, such as insecurity, uncertainty, and a sense of not being part of the implementation process. [14,42,43].

The innovation elements of the cardiac TH program, when implemented on a large scale and across hospitals, municipal health centers, and a call center, have not been previously published. We did identify a study by Frederix et al [21] on the TH for cardiac patients in Belgium. However, this study was not conducted on a significant scale, nor were the organizational issues explored or evaluated.

### Strengths and Limitations

A case study is circumscribed by the possibilities for generalization [44]. A triangulation of data collection techniques has been used in order to collect sufficient and varied data and to ensure validation of different perspectives. A longitudinal study of the cooperation among health professionals across sectors would have strengthened the results, as would a larger randomized control trial study with more patients enrolled so that health care professionals would gain more experience working with TH. We are aware that a potential limitation of this study is that it reflects specific elements of the Danish context, where all health care services are public and free of charge.

### Conclusions

The TTP was a new, innovative cardiac TH program that was tested on a large scale across hospitals, health care centers, and municipalities. Assessments of the cooperation between the health care professionals showed that the Teledialog program and its associated technologies helped to integrate workflows, created a joint clinical practice, and fostered a common sense of purpose among the organizations. Interorganizational cooperation was improved, and fragmentation of tasks reduced, resulting in a significant benefit for the patients and satisfaction for the health professionals. Future research should focus on longitudinal case studies for assessing interorganizational cooperation between health care professionals.

## Appendix

Multimedia Appendix 1 Video of the Teledialog Project.

Multimedia Appendix 2 Observation guide.

Multimedia Appendix 3 Interview guides.

## Abbreviations

CR: cardiac rehabilitation
CTH: cardiac telerehabilitation
CVD: cardiovascular disease
PHR: personal health record
TH: telerehabilitation
TTP: Teledialog Telerehabilitation Program
