# Supplementary material for: The Use of Telerehabilitation Technologies for Cardiac Patients to Improve Rehabilitation Activities and Unify Organizations: Qualitative Study
Source: JMIR Rehabil Assist Technol. 2018 Nov 19;5(2):e10758. doi: 10.2196/10758 (PMC6277831; doi:10.2196/10758)
Supplement: Multimedia Appendix 2 [file rehab_v5i2e10758_app2.pdf]

Appendix 2: Observation guide

Themes for observations

1. Interaction between healthcare professionals across sectors
    a. Dialogue on specific patient cases
    b. Communication between professional staff (controversies, meetings, e-rehabilitation plan)
    c. Verbal and non-verbal communication
    d. Knowledge-sharing within the interdisciplinary cardiac team (meetings, e-rehabilitation plan)

2. Interaction between patients and healthcare professionals
    a. Dialogue with patient (phone, e-rehabilitation plan)
    b. Interaction (verbal/non-verbal) between patient and healthcare professional at out-patient clinic
