# Supplementary material for: The Use of Telerehabilitation Technologies for Cardiac Patients to Improve Rehabilitation Activities and Unify Organizations: Qualitative Study
Source: JMIR Rehabil Assist Technol. 2018 Nov 19;5(2):e10758. doi: 10.2196/10758 (PMC6277831; doi:10.2196/10758)
Supplement: Multimedia Appendix 3 [file rehab_v5i2e10758_app3.pdf]

Appendix 3: Interview guides, phases I and II

Phase I (November to December 2012)

1. Mutual presentation
2. Can you describe your experience with cardiac rehabilitation?
3. What are the possibilities and challenges within interprofessional co-operation across sectors in the area of cardiac rehabilitation? (meetings, written communication between professionals and with patients)
    - Communication on specific patients' cases
    - Relations between professionals
    - Task solving
    - Knowledge-sharing between professional groups
4. Other issues for consideration or reflections on inter-organizational co-operation within cardiac rehabilitation?

Phase II (December 2013 to January 2014)

1. Mutual presentation
2. What have you experienced with co-operation within the Teledialog Telerehabilitation program?
    - Communication on specific patients' cases
    - Relations between professionals
    - Task solving
    - Knowledge-sharing between professional groups
3. What are the advantages and disadvantages of the e-rehabilitation plan across sectors between healthcare professionals?
    - Workflows
    - Communication
    - Coordination on tasks
    - Relations
    - Controversies between healthcare professionals
4. Other emerging issues for consideration or reflections on inter organizational co-operation within telerehabilitation?
